# Intradural Extramedullary Solitary Fibrous Tumor of the Thoracic Spinal Cord

**DOI:** 10.7759/cureus.18613

**Published:** 2021-10-08

**Authors:** Zachary T Olmsted, Joanna Tabor, Omer Doron, Hossein Hosseini, Daniel Schneider, Ross Green, Samuel J Wahl, Daniel M Sciubba, Randy S D'Amico

**Affiliations:** 1 Neurological Surgery, Northwell Health, New York, USA; 2 Pathology and Laboratory Medicine, Northwell Health, New York, USA; 3 Neurological Surgery, Lenox Hill Hospital, New York, USA

**Keywords:** neoplasm, primary tumor, extramedullary, intradural, thoracic spinal cord, hemangiopericytoma, solitary fibrous tumor

## Abstract

Solitary fibrous tumors (SFTs) are rare soft tissue neoplasms that can impact the central nervous system (CNS). SFTs comprise <1% of all primary CNS tumors. Here, we describe a rare case of intradural, extramedullary SFT arising within the thoracic spine that was treated with surgical resection. Histological features were evaluated and revealed a highly cellular tumor with positive expression of BCL2, CD34, CD99, and STAT6 proteins that are consistent with a diagnosis of SFT. We discuss the use of surgical intervention for long-term disease control of spinal SFT and evaluate the role of postoperative radiation therapy in management strategies. Lastly, we review the literature reports of intradural, extramedullary SFTs in the thoracic spine. The importance of molecular characterization by histopathology to properly determine diagnosis and prognosis is emphasized.

## Introduction

A meningeal solitary fibrous tumor (SFT) and hemangiopericytoma were previously described as separate entities having common genetic and molecular properties [1]. These rare, dural-based neoplasms were first described in 1942 and represent <1% of primary central nervous system (CNS) tumors. Recently, the WHO classification of these neoplasms was revised to unify the separate diagnoses under the common term "SFT" in order to reflect the soft tissue nomenclature [2]. Genetically, both intracranial and extracranial SFTs have an inversion at the 12q13 locus resulting in a fusion of the *STAT6* and *NAB2* genes. Diagnosis is typically made based on the nuclear localization of STAT6 that results from this fusion event and is a surrogate marker used in place of whole-genome sequencing [3]. The additional co-expression of CD44 and CD99 is also a common molecular finding. Despite similar molecular profiles, distinct features related to SFT aggressiveness and recurrence are seen in the CNS versus peripheral soft tissues. Within the CNS, SFT is an aggressive, malignant neoplasm with a pronounced capillary-derived vascular component that is distinct from peripheral soft tissue masses. Despite the revised nomenclature, CNS SFTs continue to be graded according to mitotic count per 10 high-power fields (hpf) as grade I, grade II (<5 mitoses), or grade III (>5 mitoses) [1,2]. Origination of SFT within the spine is exceedingly rare. In fact, these account for only 10% of all CNS-localized SFTs while the remainder originates rostrally to the spinal canal [1]. Unfortunately, as with their intracranial counterparts, extramedullary spinal SFTs exhibit high recurrence rates and a significant propensity for metastasis.

The current best treatment practices for SFT consist of safe, gross total resection (GTR) regardless of the site of origin. Although adjuvant radiotherapy (RT) has been proposed to reduce recurrence risk, survival outcomes remain mixed [4-6]. Accurate diagnosis remains critical to guide treatment goals. For intradural, extramedullary SFTs, recurrence has been observed in approximately 44% of cases with metastases in up to 20% of patients [7]. Interestingly, the time to recurrence of spinal SFT is shorter than for intracranial lesions, usually occurring within the first five years after resection of the primary mass. However, mortality rates of intracranial SFTs are higher than for spinal SFTs [8].

Here, we present a rare case of intradural, extramedullary SFT (grade III) arising within the thoracic spine. We discuss the clinical features and review in detail the relevant histopathology. We also provide a review of the reported literature cases of intradural, extramedullary SFTs identified within the thoracic spine.

## Case presentation

**Figure 1:**
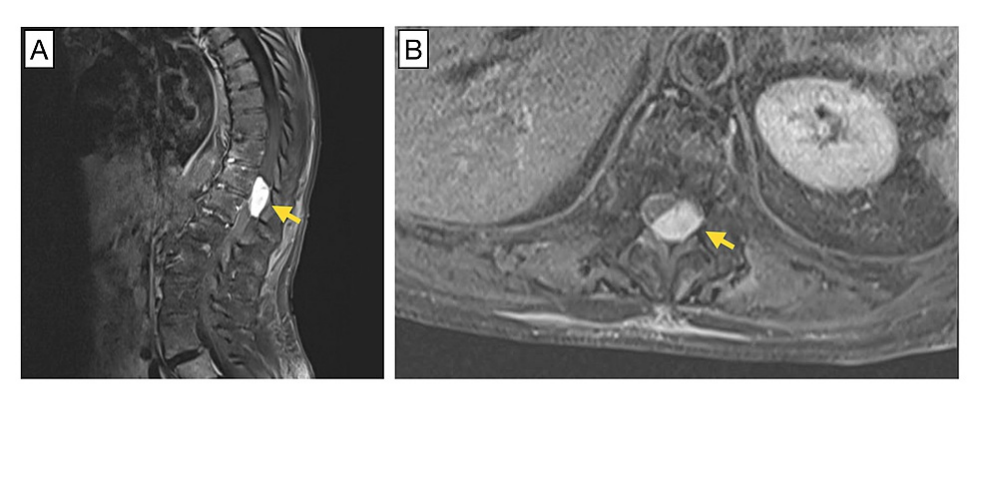
MRI of the spinal column prior to surgical resection. Sagittal (A) and transverse (B) pre-operative MRI demonstrate a contrast-enhancing 3.1-cm T11-12 left lateral mass with intradural, extramedullary localization (yellow arrows).

An 81-year-old woman with a past medical history of ulcerative colitis, hypothyroidism, and hypertension presented with lower back pain progressing to paraparesis and decreased sensation in the lower extremities over the course of eight months. At presentation, she had already lost the ability to ambulate and had difficulty controlling bowel movements for over two weeks. Magnetic resonance imaging (MRI) of the thoracic spine demonstrated severe spinal cord compression due to a contrast-enhancing, intradural, extramedullary lesion at the T11-12 level measuring 3.1 cm in the greatest dimension (Figure 1). The intradural mass was lobular, resembling a soft tissue mass, and was seen along the left lateral and posterior margins of the cord. Soft tissues outside of this site were unremarkable. The patient was transferred to our hospital and underwent resection of the intradural mass for both therapy and diagnosis.

**Figure 2:**
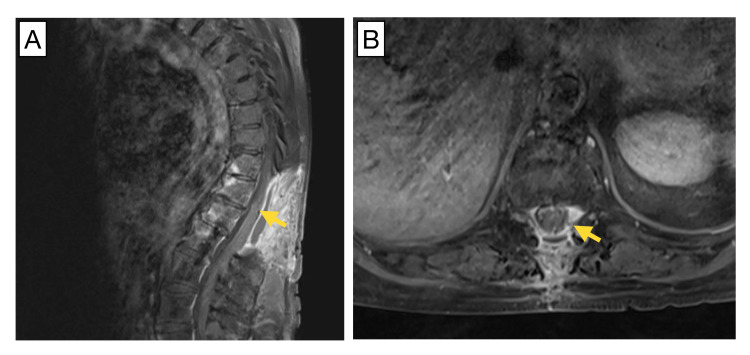
Postoperative MRI of the spinal column. Sagittal (A) and transverse (B) postoperative MRI demonstrate excision of the left lateral mass (yellow arrows).

Standard laminectomies extending from T10-12 were performed and assisted with concomitant intraoperative neuromonitoring. The tumor was observed to arise from the left T11 nerve root entry zone with notable pial infiltration into the left posterolateral aspect of the spinal cord at the root entry zone. The tumor had completely invaded and replaced the T11 nerve root, which was ultimately sacrificed during resection, leaving a small amount of deep foraminal tumor laterally. Neuromonitoring remained stable throughout the procedure. Post-operative MRI demonstrated the absence of the contrast-enhancing mass but with diffuse STIR and T2 hyperintensity reflecting edema and normal post-laminectomy findings (Figure 2). The patient was neurologically stable post-operatively with some improvement in motor strength and was discharged for rehabilitation.

**Figure 3:**
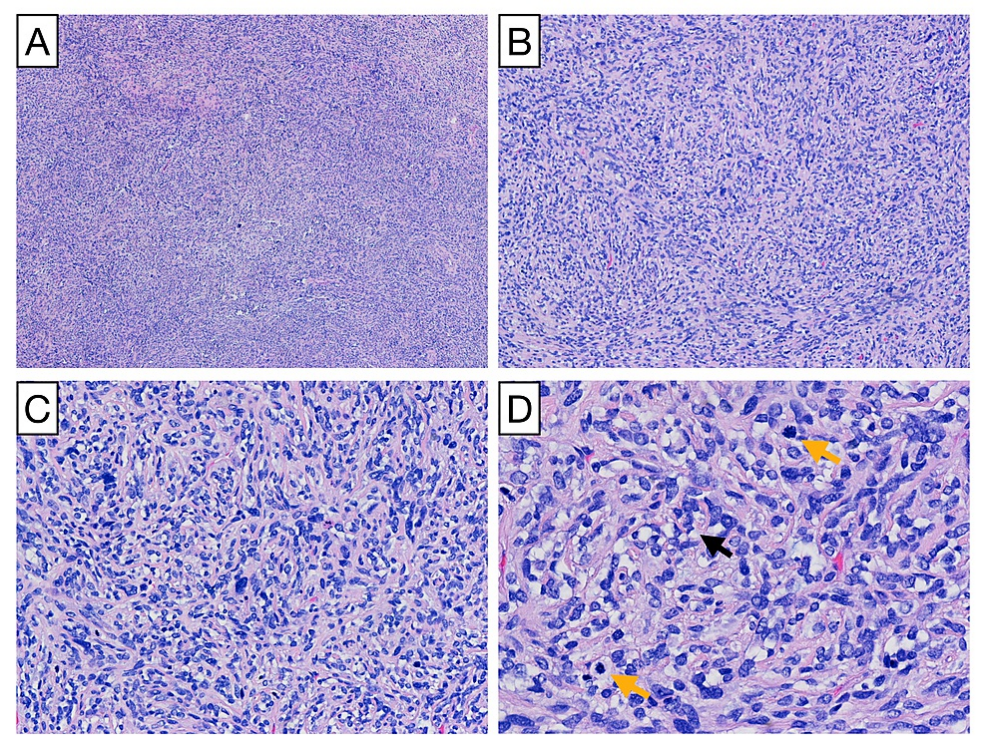
Solitary fibrous tumor histopathology. Cellular spindle cell tumor with marked nuclear pleomorphism and high mitotic activity (hematoxylin-eosin): 40× (A), 100× (B), 200× (C), and 400× (D). Black and yellow arrows in (D) denote nuclear pleomorphism and mitotic figures, respectively.

**Figure 4:**
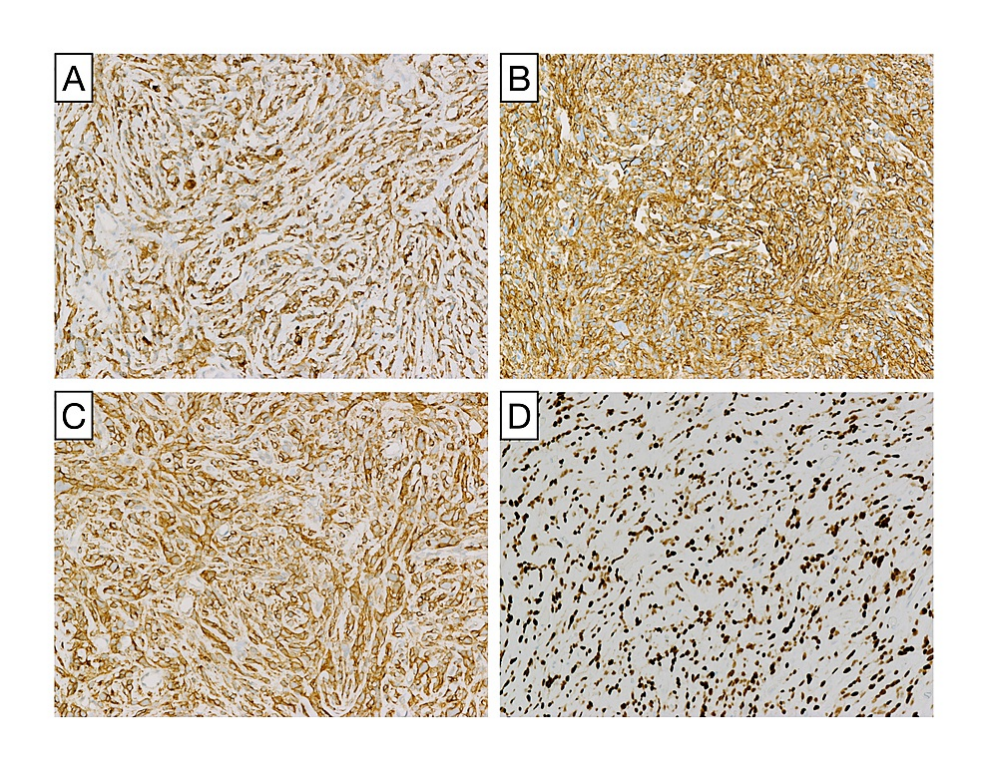
Immunohistochemistry validation of solitary fibrous tumor. Tumor cells express biomarkers BCL2 (A), CD34 (B), CD99 (C), and the nuclear marker STAT6 (D) indicating SFT. All images are 200× magnification.

Gross anatomical analysis of the specimen identified a homogenous white-pink rubbery lesion. The histopathological analysis further identified a spindle cell neoplasm composed of closely packed cells with marked nuclear pleomorphism and up to 8 mitoses per 10 hpf (Figure 3). Spindle cells were arranged haphazardly with scattered, inconspicuous thin-walled blood vessels that are characteristic of SFTs. Frank necrosis was not present within the tumor. Immunohistochemical analysis demonstrated cytoplasmic staining of BCL2, robust expression of CD34 and CD99, and nuclear expression of STAT6. These findings are highly sensitive and specific for SFT (Figure 4). The final diagnosis of SFT grade III was made based on immunohistochemical profiling, high cellularity, nuclear pleomorphism, and high mitotic index.

**Figure 5:**
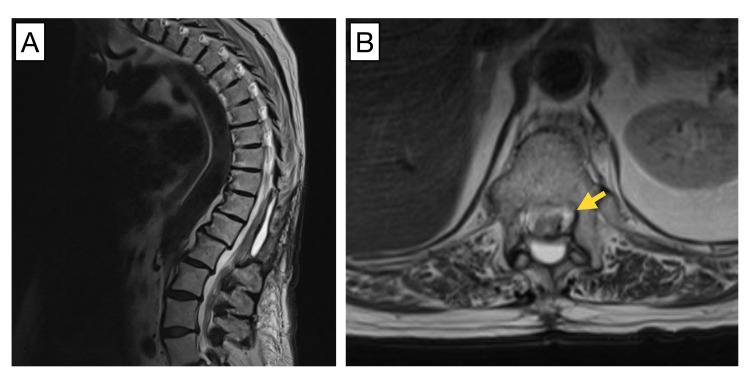
Repeat MRI at three months follow-up. Follow-up MRI showed a stable nodular focus of enhancement at the T11-12 level extending into the proximal left neural foramen (yellow arrow). Sagittal (A) and transverse (B) images are shown.

At a three-month follow-up, the patient had significant symptom improvement and was able to ambulate with a walker at home. MRI showed an unchanged nodular focus of enhancement at the T11-12 level extending into the left proximal neural foramen representing known residual tumor burden (Figure 5). Interestingly, despite the aggressive histopathological phenotype, this lesion remained stable on serial imaging at six months.

## Discussion

Here, we present a rare case of a primary intradural, extramedullary WHO Grade III SFT arising within the thoracic spine in an 81-year-old woman. While SFT arising within the spine has been described previously, intradural extramedullary tumors remain rare with only 46 cases in the thoracic spine as identified in 28 separate retrospective studies (Table 1) [7]. Of these, 64.9% were male and 35.1% were women. The median age was 50 years with a range of 12-82 years. The case presented here is only the second case of thoracic intradural extramedullary SFT reported in a woman aged 80 years or more. The diagnosis of SFT was made based on gross morphology, expression of CD34/CD99, and nuclear expression of STAT6. Since nuclear expression of STAT6 is a distinguishing feature of the characteristic *NAB2-STAT6* fusion, this finding is highly indicative of a diagnosis of SFT versus other neoplasms (e.g., S100+ schwannoma) [3]. Immunostaining for S100+ was negative. The SFT was designated grade III due to high mitotic index (up to 8 mitotic figures per 10 hpf) and marked nuclear pleomorphism was appreciated in dense spindle cells. Patients with SFT outside of the nervous system are generally risk-stratified according to the updated Demicco criteria incorporating patient age (≥55 years), tumor size (≥15 cm), mitotic activity (≥4/10 hpf), and necrosis (>10%) [9,10]. It remains undetermined whether the Demicco criteria can be applied to SFTs arising within the spinal cord (e.g., tumor size ≥15 cm). However, consistent with the general Demicco criteria, a recent study by Fritchie et al. demonstrated that the extent of surgery, use of radiotherapy and/or chemotherapy, mitotic rate, and necrosis were significant in predicting progression-free survival and disease-specific survival for meningeal SFT [11]. We did not observe necrosis in the resected 3.1 cm mass, indicating a lower risk stratification for metastasis. However, given that the patient is ≥55 years with a high mitotic index, close follow-up is indicated.

**Table 1:** Reported cases of intradural, extramedullary solitary fibrous tumor in the thoracic spine

Study	Total cases	Thoracic cases	Thoracic level	Age	Sex	Treatment	Recurrence (Yes/No)	Metastatic (Yes/No)	Follow-up, recurrence-free survival (y)
Pitlyk et al. [22]	3	1	T8	39	M	GTR	Yes	No	10
Malek et al. [23]	1	1	T7-8	33	M	GTR	Yes	No	N/K*
Vorster et al. [24]	1	1	T2-3	51	M	GTR	No	No	0.58
Kurtkaya et al. [25]	1	1	T3	73	F	GTR	No	No	1
Pizzolitto et al. [26]	2	1	T7-8	36	M	GTR	No	No	1.5
Jallo et al. [27]	4	2	T2-3	37	F	GTR	No	No	5
			T5	59	M	GTR	No	No	4.8
Kashiwazaki et al. [28]	1	1	T4-6	31	M	GTR	No	No	3
Zhao and Zhao [29]	23	9	N/K	N/K	N/K	GTR	No	No	4.7
Chou et al. [30]	1	1	T10	80	M	GTR	No	No	3
Moscovici et al. [31]	1	1	T9-10	20	M	GTR	No	No	2
Ackerman et al. [32]	1	1	T10	58	M	GTR	No	No	N/K
Brigui et al. [33]	2	1	T6-7	69	M	GTR	No	No	N/K
Torigoe et al. [34]	1	1	T6-7	51	F	GTR	Yes	No	5
Shirzadi et al. [19]	4	1	T9-10	57	M	GTR + RT	No	No	3
Liu et al. [35]	26	2	T5-7	19	F	SRT + RT + CT	Yes	No	25
			T12-L1	23	F	GTR + RT	Yes	No	6
Kaur et al. [36]	1	1	T9	16	M	GTR + RT	No	Yes	5
Türk et al. [37]	2	1	T9-10	15	F	GTR	No	No	N/K
Das et al. [38]	5	1	T11-L1	12	M	GTR + RT	No	No	0.75
Chew et al. [39]	1	1	T9	80	M	GTR	No	No	1
Yi et al. [40]	11	1	T4-S1	64	M	GTR	N/K	No	N/K
Wang et al. [41]	16	7	T3-4	21	M	GTR	No	No	5.58
			T3-4	57	M	GTR + RT	No	No	8
			T4	49	M	GTR	No	No	2.91
			T6	37	F	GTR	Yes	No	1.83
			T8	43	F	GTR + RT	Yes	No	4.08
			T9	33	F	GTR + RT	No	No	5.8
			T11-12	40	F	STR + RT	No	No	3.5
Fujita et al. [42]	1	1	T7	50	M	GTR	No	No	N/K
Louis et al. [43]	1	1	T9-10	82	F	GTR	No	No	N/K
Paeng [44]	1	1	T5-6	57	M	GTR + RT	No	No	2
Tomomatsu et al. [45]	1	1	T9	68	F	GTR	No	No	3
Kim et al. [46]	1	1	T1-2	64	M	GTR	No	No	1
Okubo et al. [47]	10	3	N/K	55	M	PR	No	No	4.17
			N/K	49	M	PR	Yes	No	2.92
			N/K	62	M	GTR	No	No	2.5
This study	1	1	T11-12	81	F	GTR	No	No	0.5

SFT and hemangiopericytoma were previously distinguished as separate diagnoses with similar molecular profiles, but the term hemangiopericytoma has been retired. All CNS tumors of this kind are now classified as SFTs [2]. In the CNS, there is typically a significant and pronounced vascular component that was in part responsible for the previous classification as hemangiopericytoma. SFT in the CNS is aggressive with the risk of local recurrence and metastasis. While SFT neoplasms represent <1% of all CNS tumors, the majority of these (90%) occur intracranially above the tentorium cerebelli and 10% occur in the spine. For both intracranial and spinal lesions, surgical resection for the symptomatic disease is the first choice of treatment and should be attempted whenever possible [12,13]. Surgical resection must be planned appropriately in order to minimize collateral damage to critical neurovascular structures and hemorrhage. RT has been shown to be beneficial following subtotal resection (STR) in limiting disease progression. For intradural, extramedullary tumors, recurrence even after GTR occurs in approximately 44% of cases [7]. Time to recurrence of SFT is higher in spinal versus intracranial tumors and usually occurs within the first five years after resection. Despite this, intracranial tumors have a higher mortality rate [8].

Spinal SFT can be classified into four anatomic locations for surgical planning, similar to other spinal tumors. This includes vertebral (osseous), paravertebral, spinal canal, and mixed location. For lesions within the spinal canal, three subtypes exist that are extradural, intradural extramedullary, and intradural, intramedullary. Spinal SFT typically presents with focal deficits due to mass effect at the corresponding vertebral level. In the case presented here, progressive tumor growth and compression of the spinal cord at T11 resulted in paraparesis and decreased sensation bilaterally in the lower limbs over an eight-month period.

SFT is often misdiagnosed radiographically given the similarities to other spinal lesions on imaging and lack of radiographic pathognomonic findings. Common misdiagnoses include meningioma, schwannoma, hemangioma, metastases, and primary bone malignancies. Therefore, a definitive diagnosis is typically made by pathology after resection of the mass.

CNS SFT is graded based on the mitotic index. Five mitoses per 10 high power fields (hpf) represents the threshold between WHO Grade II and Grade III disease [1]. The mitotic index, in this case, was up to 8 mitoses per 10 hpf, consistent with a grade III tumor. We observed STAT6 nuclear staining that is a finding highly sensitive and specific for SFT due to the inversion at the 12q13 locus. We also observed robust CD34 and CD99 biomarker expression, corroborating the diagnosis of SFT. Additional features included high cellularity with spindle cell morphology and marked nuclear pleomorphism. Hemorrhage and necrosis may occur in grade III lesions due to the vascular nature of the tumor. However, this was not apparent on this pathologic specimen.

The question of whether spinal cord SFT is more benign with respect to intracranial counterparts remains unknown. In this case, we observed relatively benign tumor behavior despite common genetic precursor variants with previously reported aggressive SFT CNS neoplasms [7]. One hypothesis is that spinal cord tumors produce symptoms at an earlier stage given the confined space within the spinal column and that early resection limits disease progression. This may be evaluated in future studies comparing tumor size by location at the time of resection. Mortality could be higher in intracranial disease due to propensity for recurrence within regions involving numerous neuroanatomical structures or a prolonged timeframe from tumor development to detection. An alternate hypothesis is that the vascular SFT component is reduced in spinal versus intracranial neoplasms. While not currently available in the literature, a side-by-side comparative analysis of vascular characteristics of spinal versus intracranial SFTs may provide insights into pathogenesis and prognosis. The time to recurrence of spinal SFT has been previously reported to be approximately five years. Therefore, the latest follow-up timepoint in this case (six months) may be too early to accurately characterize ultimate tumor behavior. Logarithmic tumor growth patterns can underlie the rapid expansion of 3D tumor volume. Extended follow-up is therefore warranted and may be related to modified Demicco criteria for SFT.

Given the rare occurrence of SFT spinal neoplasms, no prospective, randomized control trials are available. Our approach and outcome support the current standard of care for SFT that includes safe maximal resection. In general, we support aggressive resection for optimal treatment, but acknowledge that this must be evaluated on a case-by-case basis. Progression-free survival and overall survival remain unknown. For this 81-year-old patient, a residual tumor was left in the foramen since definitive diagnosis at the time of operation remained unclear on frozen section analysis. Since definitive diagnosis was not known at this time, frozen section analysis clearly indicating more aggressive cellular phenotypes may have indicated more aggressive resection. This elderly patient was already severely functionally affected prior to surgical resection. Therefore, it was reasonable to offer a smaller but effective resection in the face of the unknown diagnosis. In a younger patient, it may have been indicated to unroof the foramen and commit to a small segment fusion in the case that the frozen section exhibited evidence of high-grade aggressive disease. The current consensus indicates that stereotactic radiosurgery can be useful to prevent disease progression and reduce recurrence, thereby lengthening progression-free survival time [14]. However, adjuvant RT has not been consistently shown to improve overall survival time [6,15]. Nevertheless, RT should be considered for SFT cases that undergo STR instead of GTR and for high-grade neoplasms with aggressive phenotypes [16]. Median survival time and recurrence rates have been studied for intracranial SFTs managed by GTR versus STR [17,18]. Similar data for spinal cord SFT is lacking and insufficient to draw meaningful conclusions [19]. One spinal cord study by Souyer et al. reported that five-year local control rates were higher for patients treated with GTR (84%, 17 patients) versus STR (38%, 2 patients) [20]. The earliest recurrence rate occurred in one of the patients who underwent STR plus adjuvant RT, although no recurrence was seen with intramedullary involvement and survival was 100% at three-years follow-up in that study. In general, GTR is the preferred method of treatment versus STR, even when STR is combined with adjuvant RT. Future studies of SFT mechanisms should include a detailed investigation into the *NAB2-STAT6* fusion events using genome sequencing with a focus on exon and intron breakpoints. Variation in exon fusions has been linked to recurrence rates in conventional SFTs versus previously described hemangiopericytomas that exhibit more aggressive phenotypes [21].

We reviewed the literature for cases of intradural, extramedullary SFT in the thoracic spine and provided this in Table 1. This table demonstrates a distinct absence of metastatic disease related to the intradural, extramedullary SFT subtype in the thoracic spine that may be useful to inform future management strategies.

## Conclusions

SFT neoplasms of the CNS and spinal column are rarely reported in the literature. Because of low incidence rates, it is a challenge to construct prospective studies to elucidate the molecular nature and optimal management of spinal SFT. It is imperative to determine the effectiveness of adjuvant RT and chemotherapy to maximize survival rates, therapeutic outcomes, and reproducibility. This is particularly important as factors portending a more aggressive phenotype remain unknown. In this report, we describe a rare case of intradural, extramedullary SFT of the thoracic spine, the second reported case in a female ≥80 years. Tumor cells expressed STAT6 re-localized to the nucleus that is a sensitive and specific surrogate biomarker for *NAB2-STAT6* gene fusion, a characteristic feature of SFT. Our findings support the maximum possible GTR as an appropriate surgical intervention in accordance with the current standard of treatment, although adjuvant radiotherapy can be considered after STR to hamper disease progression. High-risk features including age ≥55 years, high mitotic index, and the presence of tumor necrosis warrant close follow-up. By reviewing reported cases of intradural, extramedullary SFT in the thoracic spine, we identify a distinct lack of metastatic disease in this subtype that may inform future management strategies. However, recurrence remains common. Future studies centered around mechanisms driving faster SFT recurrence in the spine versus the more aggressive intracranial tumors will be essential to understanding the disease process to optimize treatment. A comparative analysis of vascular characteristics of spinal versus intracranial SFTs may provide insights into pathogenesis and prognosis. Such studies should include a detailed investigation into different *NAB2-STAT6* fusion events using whole-genome sequencing since different exon fusion sites have been linked to recurrence rates and aggressive behavior in conventional SFTs.
